# Nanocellulose-Based Adsorbents for Heavy Metal Ion

**DOI:** 10.3390/polym14245479

**Published:** 2022-12-14

**Authors:** Rongrong Si, Junwen Pu, Honggang Luo, Chaojun Wu, Gaigai Duan

**Affiliations:** 1 Beijing Key Laboratory of Lignocellulosic Chemistry, Beijing Forestry University, Beijing 100083, China; 2 State Key Laboratory of Biobased Material and Green Papermaking, Qilu University of Technology (Shandong Academy of Sciences), Jinan 250353, China; 3 Jiangsu Co-Innovation Center of Efficient Processing and Utilization of Forest Resources, International Innovation Center for Forest Chemicals and Materials, College of Materials Science and Engineering, Nanjing Forestry University, Nanjing 210037, China

**Keywords:** nanocellulose-based adsorbents, chemical modification, heavy metal ions, assembling

## Abstract

Heavy metal ions in industrial sewage constitute a serious threat to human health. Nanocellulose-based adsorbents are emerging as an environmentally friendly material platform for heavy metal ion removal based on their unique properties, which include high specific surface area, excellent mechanical properties, and biocompatibility. In this review, we cover the most recent works on nanocellulose-based adsorbents for heavy metal ion removal and present an in-depth discussion of the modification technologies for nanocellulose in the process of assembling high-performance heavy ion adsorbents. By introducing functional groups, such as amino, carboxyl, aldehyde, and thiol, the assembled nanocellulose-based adsorbents both remove single heavy metal ions and can selectively adsorb multiple heavy ions in water. Finally, the remaining challenges of nanocellulose-based adsorbents are pointed out. We anticipate that this review will provide indispensable guidance on the application of nanocellulose-based adsorbents for the removal of heavy metal ions.

## 1. Introduction

Nowadays, heavy metal ions have become the most serious problem in water environment due to their toxicity and incompatibility, which cause bad environmental problems and threaten human health [1]. Excessive intake of heavy metal ions can cause body damage and even death through afflictions, such as, Minamata disease in Japan [2] caused by the excessive intake of organic mercury (Hg), lung or gastrointestinal tract disease caused by the accumulation of Cd^2+^ [3], and Alzheimer’s and Parkinson’s diseases [4] caused by the excessive intake of Fe^3+^ and Al^3+^. Furthermore, the increase in nuclear power plants leads to many radioactive heavy metal pollutants, such as Cs137, Pu239 and U238 [5] (Table 1). To date, it remains difficult to eliminate such ionic pollutants from heavy metals.

Many methods exist to solve the problem of heavy metal ion pollution in wastewater, including chemical precipitation, ion exchange, ultrafiltration, flocculation, electrodialysis, adsorption, reverse osmosis, and more [6]. Among these methods, adsorption is very popular due to its high removal efficiency, flexibility in the design, and low cost [7]. Adsorbents generally consist of activated carbon, clay, biochar, and polymers [8]. Although these adsorbents have high adsorption capacities for certain heavy metal ions, they present drawbacks, such as, undesired non-biodegradability, high energy costs for preparation or regeneration, and secondary pollution. Therefore, at present it remains highly desirable to find an excellent means of heavy metal ion adsorption and easily bio-adsorbent regeneration for green/sustainable development.

**Figure 1:**
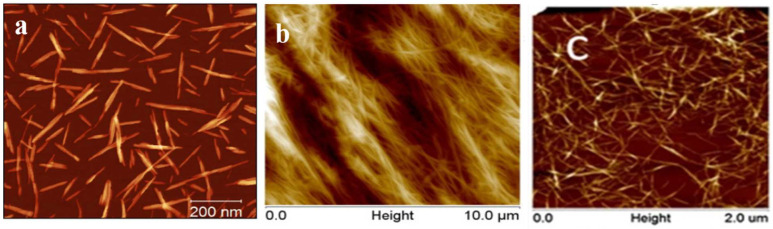
Atomic force microscope (AFM) images of (**a**) CNC, (**b**) BNC, and (**c**) CNF. Adapted with permission from [29]. Copyright 2021 Springerlink. Adapted with permission from [30]. Copyright 2021 SpringerLink. Adapted with permission from [31]. Copyright 2016 Elsevier.

Cellulose is a linear biopolymer formed by glucose units connected by β-1,4-glycosidic bonds, and mainly exists in plants, animals, algae, and fungi [9,10,11,12,13,14,15]. Nanocellulose (NC) is a cellulosic material with at least one dimension within the nanometer size. Depending on its cellulose source, processing conditions, size, function, and preparation methods, it can be classified in three categories (cellulose nanofibrils (CNFs) [16], bacterial nanocellulose (BNCs) [17] and cellulose nanocrystals (CNCs) [18]). Acid hydrolysis results in nanometer-long and highly crystalline rod-like fragments, referred to as CNCs. Mechanical shearing techniques disintegrate cellulose fibers into their substructural nanoscale units, resulting in CNFs, which are typically longer, being micrometric in length. BNC is produced through a bottom-up approach using cultures of bacteria to synthesize the material (Figure 1). To date, NC has been applied in oil-water separation [19], filter materials [20], sensing [21,22,23,24], capacitors [25], bio-scaffolds [26], and drug delivery [27], and has been explored in the fields of heavy metal ion removal as well [28].

**Figure 2:**
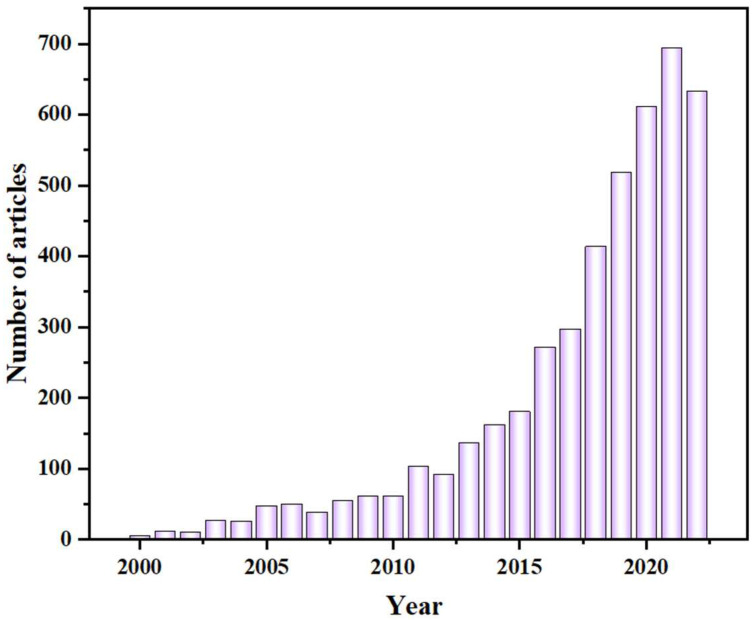
Total number of articles on cellulose adsorbents.

NC-based adsorbents have recently become more and more popular, nanocellulose has a high specific surface area, excellent mechanical properties, and good biocompatibility, making especially suitable for heavy metal ion adsorbent assembly [32,33,34]. The total number of relevant articles on cellulose adsorbents and the keywords used in their description (eliminating redundant searches) are presented in Figure 2. Adsorbents using cellulose have been described for a wide range of applications. However, as NC is challenged by intrinsic hydrophilicity and inferior heavy metal ion adsorption sites [35], it is necessary to directly/indirectly introduce key functional groups in the NC or assemble an excellent architecture in order to enhance the heavy metal ion adsorption of NC-based adsorbents.

It has been reported that the adsorption properties of NC for heavy metal ions are superior to those of macro- and microfibrillar cellulose [36,37]. In order to improve the binding sites of NC for heavy metal ions, it is necessary to introduce a number of key functional groups into the NC. With the exception of NC functionalization, methods for assembling nanocellulose-based adsorbents are very important for improving their maximum heavy metal ion adsorption capabilities. It has great application potential as a green base for adsorbent materials of aerogels, hydrogels [38], films [39], etc. Nanocellulose-based adsorbents have gradually become an environmentally friendly and appealing material for heavy metal ion removal.

Qiao et al. [28]. mainly reviewed the surface modification of nanocellulose-based adsorbents regarding the adsorption of heavy metal ions and dyes, and summarized a number of adsorption mechanisms. Kose et al. [40] described the methodologies under current use for such designs and provides a systematic overview of these technologies to promote more focused research in the future for nanocellulose-based adsorbent materials. Salama et al. [41] provided an overview of nanocellulose requirements concerning emerging nanotechnologies in wastewater treatment and purification, i.e., adsorption, absorption, flocculation, photocatalytic degradation, disinfection, antifouling, ultrafiltration, nanofiltration, and reverse osmosis. In this review, we first summarize the functionalization of NC in the molecular range, including oxidation, esterification, etherification, cationization, etc., and summarize the adsorption performance of the adsorbance for heavy metal ions. We then present the most recent works on nanocellulose-based adsorbents and highlight in depth the functionalization of nanocellulose and assembling/composing technologies for nanocellulose-based adsorbents. Finally, we conclude with perspectives on the challenges and opportunities that remain for nanocellulose-based adsorbents.

## 2. Functionalized Modified Nanocellulose Adsorbents for Heavy Metal Ions

There are three hydroxyl groups on each cellulose glucose ring, the hydroxyl groups on C2 and C3, and the primary hydroxyl groups on C6. Their response capacity is different because of their different positions. The secondary hydroxyl groups are larger than the primary alcohols, and etherification, esterification, oxidation, graft copolymerization, and other reactions may occur. The combination of biotechnology and nanotechnology provides a new and green way of solving the old problems. To improve the heavy metal adsorption sites and affinities of NC, there are a lot of emerging technologies for NC modification, mainly containing the introduction of carboxyl, carboxymethyl, aldehyde, cationic, phospho-containing, and sulfur-containing groups.

### 2.1. Oxidation Reaction

**Figure 3:**
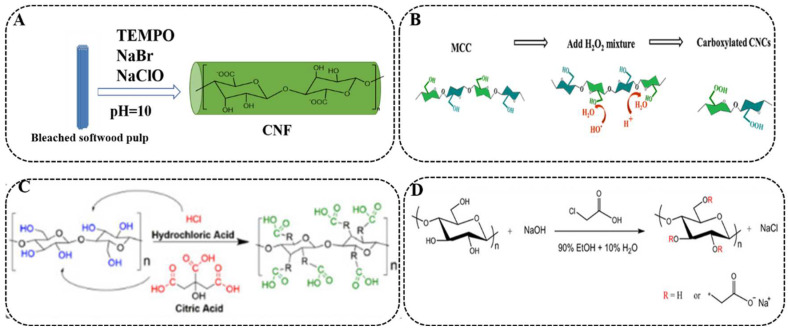
Carboxyl formation sites in various reaction systems: (**A**) TEMPO oxidation, (**B**) Fe^2+^/H_2_O_2_ oxidation, (**C**) citric acid/HCl esterification, (**D**) carboxymethylation. (Adapted with permission from [42]. Copyright 2021 SpringerLink. Adapted with permission from [43]. Copyright 2019 American Chemical Society. Adapted with permission from [44]. Copyright 2018 American Chemical Society. Adapted with permission from [45]. Copyright 2019 American Chemical Society).

TEMPO (2,2,6,6-Tetramethylpiperidine-1-oxyl)-mediated oxidation [42] has opened a field of efficient and selective chemistry for converting C6 primary hydroxyls into carboxylate groups on the surface of cellulose microfibrils under mild conditions (Figure 3A). The common TEMPO-mediated oxidation system is prepared using TEMPO, NaBr, NaClO, etc.

**Figure 4:**
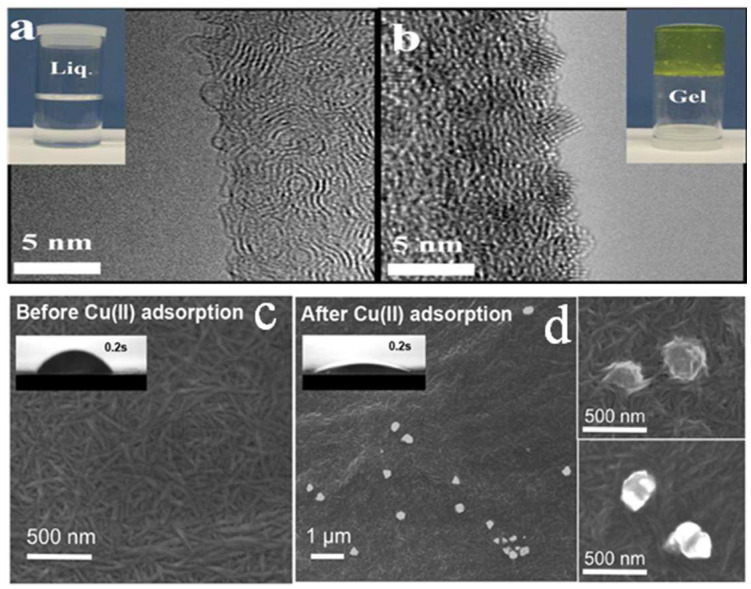
TEM image of ultrafine cellulose nanofibers (**a**) before and (**b**) after adsorption of UO_2_^2+^. SEM image of TOCNF1.5 before (**c**) and after (**d**) Cu^2+^ adsorption. Adapted with permission from [47]. Copyright 2019 Elsevier. Adapted with permission from [48]. Copyright 2016 Elsevier.

Ma et al. [46] used the TEMPO oxidation approach to prepare an aqueous suspension of 0.05 wt% ultrafine cellulose nanofiber. The average aspect ratio of the nanofiber was about 160, and its carboxyl group content was 1.4 mmol/g. Because the carboxyl group was introduced in the ultrafine cellulose nanofiber, its maximum adsorption capacity for UO_2_^2+^ was able to reach 167 mg/g, dominated by the chelation reaction between the carboxyl group and UO_2_^2+^. Furthermore, following the adsorption of UO_2_^2+^, the surface of the cellulose nanofibers was covered by metal ionic crystals. This indicates that UO_2_^2+^ could likely be used as a “cross-linker” to convert “aqueous CNF” into “gel” (Figure 4a,b).

Sometimes, nanocellulose containing different carboxylic groups can be obtained by controlling the oxidation time and the measurement of oxidizing agent. Li et al. [47] used TEMPO to oxidize hardwood kraft pulp and obtain TOCNF with different carboxyl content by adding different amounts of NaClO solutions (150–310 g) to the oxidation process. The obtained TOCNFs had the typical width of 5–8 nm and length of 1000–2000 nm, and their carboxylate contents were 0.70, 1.40, and 1.67 mmol/g (named TOCNF 0.70/1.40/1.67), respectively. The maximum adsorption capacity of TOCNF-1.40 on Cu^2+^ and Zn^2+^ reached 102.9 mg/g and 73.9 mg/g, respectively (Table 2), leading to a superfast adsorption process which can reach adsorption equilibrium within 2 min due to the high carboxyl content.

Liu et al. [48] obtained two kinds of TOCNFs from cellulose sludge with different carboxyl contents (0.6, 1.5 mmol/g) by controlling the amount of TEMPO oxidizer. At pH 8, the lowest Zeta potential of TOCNF1.5 was −70.6 mV (Table 2). Analysis of the properties of Cu^2+^ revealed that Cu^2+^ was first adsorbed on the surface by carboxyl groups, then reduced to copper (0) or assembled copper oxide nanoparticles by microprecipitation. After the adsorption of Cu^2+^, TOCNF1.5 turned superhydrophilic and copper oxide nanoparticles appeared on the surface (Figure 4c,d). The maximum adsorption capabilities of TOCNF0.6 and TOCNF1.5 for Cu^2+^ were 44.2 and 75 mg/g, respectively (Table 2).

In general, H_2_O_2_ can destroy the amorphous region of cellulose and oxidize hydroxyl groups on cellulose to create carboxyl groups. Fan et al. [43] obtained CNCs with different carboxyl contents by controlling the oxidation time (0–8 h) of MCC by Fe^2+^/H_2_O_2_ (Figure 3B). SEM images showed that the length and width ranges of the obtained CNCs were 92–140 nm and 19–23 nm, respectively. Their results indicated that the carboxyl content reached the highest value (2.2 mmol/g) with a zeta potential of –41 ± 3.3 mV and an oxidation time of 6 h (CNCs-6h). CNCs-6h showed a maximum adsorption capacity of 51.1 mg/g for Cu^2+^ due to its more numerous carboxylic acid groups (Table 2).

In the presence of excess acid, the reaction of HNO_3_ (an oxidizing agent) and NaNO_2_ produces even more HNO_2_ and releases nitrogen nitrate ions (NO^+^). The resulting nitrous ions can attack the primary hydroxyl group at C6 of cellulose to form aldehyde groups (intermediates) and carboxylic groups. Sharma et al. [49] used nitric acid/sodium nitrite to oxidize untreated jute fibers to obtain NOCNF slurry. It had very low crystallinity (35%), and had carboxyl content and surface loading of 1.15 mmol/g and −70 mV, respectively (Table 2). At a low NOCNF suspension concentration (0.23 wt%), room temperature, and pH = 7, the NOCNF was able to remove sharply Pb^2+^ ions from 50 to 5000 ppm in the initial steps, and finally its maximum adsorption capacity was as high as 2270 mg/g. In the same way, Sharma et al. [50] used nitric acid/sodium nitrite to oxidize untreated Australian *spinifex* grass to obtain NOCNF, which had low crystallinity of around 50%, a high surface charge of –68 mV, and high hydrophilicity (static contact angle 38°). The suspension (0.20 wt%) was able to remove Cd^2+^ in a large concentration range (50–5000 ppm) within a short time (≤5 min). When the Cd^2+^ concentration was 250 ppm, the removal rate was 84%. Depending on the Langmuir curve, the maximum adsorptive capacity for Cd^2+^ was up to 2550 mg/g.

Periodate is considered as a highly selective oxidant, and can convert vicinal hydroxyl groups on the C_2_ and C_3_ positions of the anhydrous glucose units (AGU) to paired aldehyde groups without significant side reactions, simultaneously cleaving the C_2_-C_3_ bond. Lei et al. [51] treated endoglucanase hydrolyzed BSKP using a grinder, the concentration of sodium periodate was adjusted to 10 g/L (DNFC-1) and 40 g/L (DNFC-2) to adjust the content of the aldehyde group. DNFC-2 had the largest aldehyde content (1.95 mmol/g), largest specific surface area (2.73 ± 0.08 m^2^/g), and a surface charge density of −(1.14 ± 0.07) × 10^−5^ eq/g. The maximum adsorption capacity of DNFC-2 for Cu^2+^ was 26 mg/g. Generally, a more negative charge on the fibrils contributes to greater electrostatic attraction to metal ions.

**Table 2:** Preparation method and properties of carboxyl-containing nanocellulose.

Sample	Method	Zeta Potential (mV)	Carboxyl Content (mmol/g)	pH	Temperature (°C)	Adsorbent Dose	Adsorption Capacity (mg/g)	Ref
CNCs-6h	Fe^2+^/H_2_O_2_ oxidation	−41 ± 3.3	2.2	/	/	/	Cu^2+^: 51.1	[43]
7-CNF	Esterification	−36 ± 3	1.18 ± 0.1	/	/	/	Cu^2+^: 45.053	[44]
CMCNF-2.7	Etherification (Carboxymethylation)	−88.3	2.7	5	Room temperature	0.3 g/L	Cu^2+^: 115.3	[45]
TOCNF1.5	TEMPO oxidation	−70.6	1.5	5	Room temperature	/	Cu^2+^: 75	[48]
NOCNF	Nitro-oxidation	−70	1.15	~7	Room temperature	0.23 wt%	Pb^2+^: 2270	[49]
CNF-MA 2%	Esterification	/	278	5.6	25	100 mg	Cu^2+^: 84.12	[52]

### 2.2. Esterification/Etherification

In addition, the combined action of hydrochloric acid/citric acid can convert hydroxyl groups on cellulose into carboxyl groups. The introduction of citric acid into the C6 primary hydroxyl group by the Fischer esterification reaction preserves the integrity of the cellulosic glycoside ring. Wang et al. [44] obtained 9-CNF, 7-CNF, and 5-CNF with different carboxyl contents according to different ratios of hydrochloric acid/citric acid (*v*/*v* = 9/1, 7/3, 5/5) from waste ginger fiber (Figure 3C). Among them, 7-CNF had the highest aspect ratio (144), the largest carboxyl content (1.18 ± 0.1 mmol/g), and the greatest negative zeta potential (−36 ± 3 mV). Afterwards, in a lyophilized CNF suspension for aerogels. The three-dimensional (3D) network structures of all aerogels were physically cross-linked by hydrogen bonding with macropore mesopores. Due to the network capture effect, load neutralization, and chain bridging of the high aspect ratio carboxylated CNF, the 7-CNF aerogel had the highest adsorption capacity for Cu^2+^ at 45.053 mg/g.

**Figure 5:**
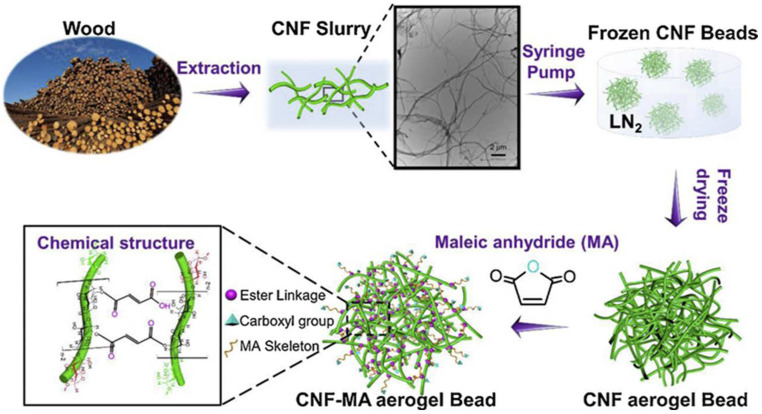
Schematic illustration of the preparation process and proposed chemical structure of the CNF-MA beads. Adapted with permission from [52]. Copyright 2019 Elsevier.

Tang et al. [52] dispersed CNF slurry in different concentrations in liquid nitrogen, rapidly froze them to form spherical beads, and freeze-dried them to obtain CNF cryogel beads. Then, carboxylated CNF/maleic anhydride cryogel beads (CNF-MA 2%) were obtained by mixing and reacting the original CNF cryogel beads and maleic anhydride (MA) solution (Figure 5). After functionalization and crosslinking of CNF cryogel beads carboxyl groups through one-step ring-opening reaction of MA, the carboxyl content reached 2.78 mmol/g and the maximum adsorption capacity for Cu^2+^ reached 84.12 mg/g (Table 2). Desorption experiments with EDTA-Na_2_ indicated that its Cu^2+^ adsorption capacity decreased from 68 mg/g to 45 mg/g after four cycles.

Yu et al. [53] obtained CNCs by hydrolyzing cotton with sulfuric acid. Subsequently, CNCs were modified with succinic anhydride and the resulting SCNCs were converted into sodic form (NaSCNCs). The maximum adsorption capacities of NaSCNCs for Pb^2+^ and Cd^2+^ were 465.1 mg/g and 344.8 mg/g, respectively, which were higher than those of SCNCs at 367.6 mg/g and 259.7 mg/g. The NaSCNCs could be efficiently regenerated with a mild saturated NaCl solution with no loss of capacity after two recyclings. The adsorption mechanism of SCNCs is a complexation process, while ion exchange is the principal mechanism for the removal of heavy metal ions by NaSCNCs.

Carboxymethylation pretreatment can introduce carboxyl groups into C_2_, C_3_ and C_6_ positions through etherification [54]. It is another alternative approach to prepare CNFs with higher carboxylate groups thanks to its non-regioselectivity (Figure 3D). Qin et al. [45] treated different amounts of wood fibers with monochloroacetic acid/sodium hydroxide and homogenization to obtain carboxymethylation CNF (CMCNFs) with different carboxyl content. Their CMCNFs showed diameters of 3.40–3.53 nm and lengths of 383.3–1210.6 nm. The carboxyl group content of CMCNF can reach up to 2.7 mmol/g (CMCNF-2.7), and the maximum zeta potential value is −88.3 mV (Table 2). Because the carboxylate group of CMCNF can capture Cu^2+^ through electrostatic attraction, ion exchange and complexation actions, its maximum adsorption capacity for Cu^2+^ reached 115.3 mg/g at pH 5.

The charges on the surface of the adsorbent determine the intensity and nature of the interactions between the adsorption sites and the adsorbents. The zero-charge point (PZC) is an important parameter that reveals the pH value of the adsorbent surface without charge [55]. The adsorption of cationic molecules is favorable when the solution pH is higher than the pH_PZC_ of the adsorbent, while the adsorption of anionic molecules is favorable at a solution pH < pH_PZC_. To sum up, the process of oxidation mentioned above controls the content of carboxyl groups by controlling the oxidant content, oxidation time, etc. In general, with more carboxyl groups, there are more anions on the surface, and the electrostatic attraction to metal ions is greater, making for a better adsorption effect.

### 2.3. Thiol Group Modified Nanocellulose

**Figure 6:**
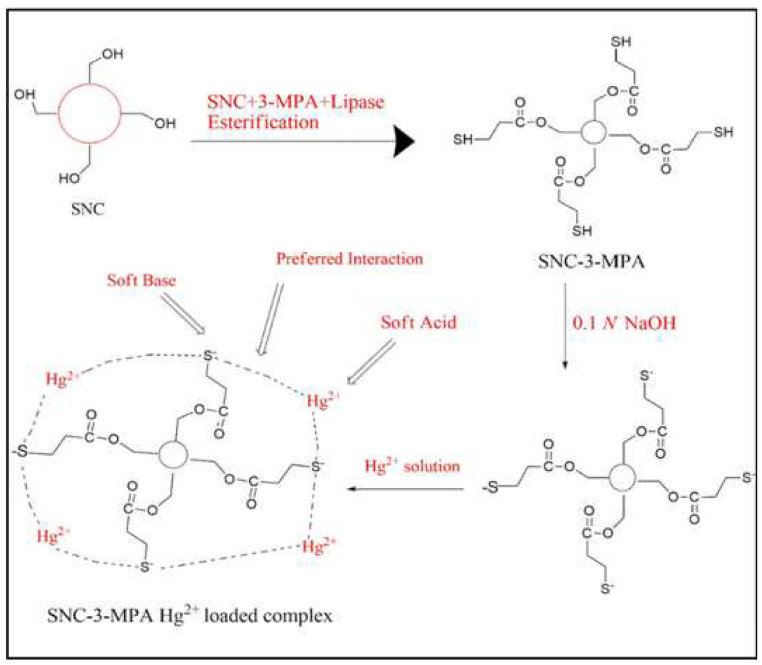
Synthetic route for SNC-3-MPA and mechanism of its Hg^2+^ ions adsorption. Adapted with permission from [58]. Copyright 2018 Elsevier.

It has been reported that thiol groups have highly selective adsorption of Hg^2+^ from wastewater [56]. According to the theory of hard−soft acid base (HSAB) [57], Hg^2+^ ions are classified as a Lewis soft acid, while thiol groups belong to the Lewis hard bases. Therefore, thiol groups tend to be preferentially complexed with Hg^2+^. Ram et al. [58] obtained spherical nanocellulose (SNC) by the treating sequences with NaOH and mixed H_2_SO_4_/HCl acid along with ultrasonication, followed by enzymatic SNC esterification with 3-mercaptopropionic acid (3-MPA) to obtain its ester derivative (SNC-3-MPA) (Figure 6). Their ^13^C-NMR (nuclear magnetic resonance) results showed that the thiol group was grafted onto the C-6-0 of the cellulose monomer rings. Because SNC has a higher specific surface area than cellulose and the presence of thiol groups has a high affinity for Hg^2+^, a removal rate of Hg^2+^ at a concentration of 100 ppm high as 98.6% could be achieved within 20 min. The maximum adsorption capacity of Hg^2+^ was 98.6 mg/g, and it could be recycled with 0.1 M HCl. The adsorbent could be regenerated and re-used for up to nine cycles, with a cumulative adsorption capacity of 404.95 mg/g.

Geng et al. [59] oxidized bamboo-derived cellulose with TEMPO to obtain TO-NFC, then subjected it to facile freeze-drying in MPTs (3-mercaptopropyltrimethoxysilane) sols to obtain flexible aerogel (TO-NFC-Si-SH). The aerogel had a high SH content of 3.33 mmol/g, its porosity reached 99.1%, the BET specific surface area was 43.57 m^2^/g, and the removal rate reached more than 92% in Hg^2+^ solution at a range of 0.01–85 mg/g. Its maximum adsorption capacity for Hg^2+^ reached 718.5 mg/g. Moreover, its adsorption capacity was nearly unchanged across a large pH range. After four adsorption/desorption experiments with 0.1 M HCl/5 wt% thiourea, its adsorption efficiency retained more than 90%. Rong et al. [60] prepared CNF-1MPTMS and CNF-2MPTMA sponges with different proportions of CNF and MPTMS (1:1/1:2) to explore the adsorption effect of Hg^2+^. The maximum adsorption capacity of CNF-2MPTMA for Hg^2+^ reached 700 mg/g, which was higher than CNF-1MPTMS (480 mg/g). After washing three times using 0.1 M aqueous disodium edetate dihydrate solution to remove Hg^2+^, the adsorption capacity of CNF-MPTMS sponges did not decrease significantly.

### 2.4. Others

Cr is one of the priority pollutants in water. Cr has two common oxidative states, of these, Cr^6+^ is highly toxic, mutagenic, and carcinogenic to the ecosystem, while Cr^3+^ is a non-toxic substance [61]. For the removal of hexavalent chromium (Cr^6+^) ions [62], most adsorbents have a higher efficiency when the pH value is less than 3; however, under neutral or alkaline conditions the removal efficiency is relatively lower.

**Figure 7:**
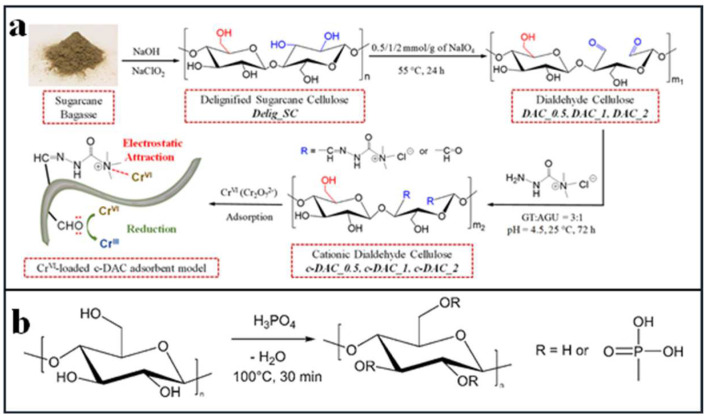
(**a**) Schematic illustration of the preparation of c-DAC adsorbent/coagulant and the corresponding adsorption mechanism for the removal of Cr(VI) from water. (**b**) Modification of cellulose nanofibrils with phosphate groups. Adapted with permission from [63]. Copyright 2020 American Chemical Society. Adapted with permission from [67]. Copyright 2016 Springer link.

Huang et al. [63] oxidized sugarcane bagasse with metaperiodate-oxidization followed by cationization using Girard’s T reagent to obtain cationic dialdehyde cellulose (c-DAC) (Figure 7a). There were a high density of quaternary ammonium groups and aldehyde groups on the surface of the c-DAC. The electrostatic attraction between the positively charged quaternary ammonium salt group and the negatively charged dichromate was the main mechanism of adsorption, and there was a strong binding affinity between the adsorbent and Cr^6+^. When adsorption reached saturation, the superficial charge on c-DAC was neutralized to form flocs of c-DAC-chromium, which could easily be removed by deincandation or low-cost gravity microfiltration. The maximum adsorption capacity for Cr^6+^ reached 80.5 mg/g, and it had stable adsorption performance across a wide pH range (2–10).

To compare the effect of CNCs and CNFs on the adsorption performance of heavy metal ions, Liu et al. [64] compared the adsorption capacity of Argentum (Ag^+^) on CNCs that were obtained by hydrolyzing sludge with H₂SO₄ and CNFs obtained from grinding. There are SO_3_^−^ groups on the surface of CNCs. The zeta potential of CNC is −44.4 mV at pH 12.09, while that of CNF is −22.7 mV under acidic conditions (pH = 1.75). The capture of heavy metal ions is accomplished by electrostatic attraction. The maximum adsorption capacities of the CNCs and CNFs for Ag^+^ reached 34.4 mg/g at pH 6.39 and 15.45 mg/g at pH 5.45, respectively.

Lignin is a conjugated polymer with a high consistency of aromatic groups that can interact with cations, moreover, the oxygen-containing groups (the hydroxyl, methoxy, and phenolic groups) are potential interaction sites of lignin for water purification [65]. Sirvio et al. [66] used DES (sulfamic acid and urea) to treat lignin-rich groundwood pulp and sawdust to obtain sulfation of sulfated wood nanofibers (SWNFs) and sulfated sawdust nanofibers (SSDNFs). As a comparison, lignin-free bleached cellulose fibers were treated in the same way to obtain sulfated cellulose nanofibers (SCNFs). The surface of the three obtained CNFs all contained sulfate ester groups. As the result of the presence of lignin, the adsorption capacity of the SWNFs and SSDNFs was increased. The maximum adsorption capacity of SWNFs for Cu^2+^ and Pb^2+^ was 158.75 and 331.2 mg/g, respectively, and the maximum adsorption capacity of SSDNFs for the two were 139.7 and 331.2 mg/g, respectively.

Mautner et al. [67] modified cellulose nanofibrils from the fiber sludge with phosphoric acid to obtain phosphorylated cellulose nanofibrils (Figure 7b), then prepared nanopapers (CNF-P) using papermaking methods. The maximum adsorption capacity for Cu^2+^ reached 19.6 mg/g through the ion exchange capture action of phosphate groups in CNF-P (18.6 ± 2.3 mmol/kg). Even after one desorption cycle using 0.1 M H_3_PO_4_, the adsorption capacity of Cu^2+^ was able to reach 19.4 mg/g.

Liu et al. [68] prepared CNC_SL_ and CNF_SL_ from cellulose sludge using hexokinase enzymes as biocatalysts and grafting the phosphate group of adenosine-5′-triphosphate (ATP) on CNF_SL_/CNC_SL_ to obtain phos-CNC_SL_ and phos-CNF_SL_ adsorbents. In contrast, nanocrystals (CNC_BE_) prepared by bioethanol were used to investigate the adsorption capacity of Ag^+^, Cu^2+^ and Fe^3+^. The results showed that the maximum adsorption capacities of Phos-CNF_SL_ for Ag^+^, Cu^2+^, and Fe^3+^ were 12,011,473 mg/g, which were all lower than the 136,117,115 mg/g of Phos-CNC_SL_. It was able to remove more than 99% of Cu^2+^ and Fe^3+^ in wastewater from the mirror making industry.

## 3. Nanocellulose/Organic Substance Composite Adsorbents for Heavy Metal Ions

Nanocellulose can be used as an adsorbent matrix/substrate, and the structure and properties can be controlled though cross-linking, additives, or assembly processes. Due to the high aspect ratio of nanocellulose, it can be entangled with other polymers to form hydrogels. In order to explore the versatility of nanocellulose-based adsorbents, adsorbents can be obtained with more functional groups in combination with other materials. Composites of two or more polymers have become a new development trend in biomaterials, as they allow certain excellent properties to be obtained that a single polymer cannot achieve [69]. Although nanocellulose has good adsorption capacity for heavy metal ions, the separation of the nanomaterials after adsorption requires high-speed centrifugation, which limits their use in large scale processes as well as their cycle performance. Consequently, their application can be improved by compounding organic or inorganic materials into aerogels, hydrogels, or other materials with 3D structure.

### 3.1. Nanocellulose/Bio-Based Organic Composites

Chitin contains polyelectrolytes and organic polymer groups and exhibits numerous nitrogen-carrying amine groups (-NH_2_) and hydroxyl groups (-OH) through several mechanisms, including chemical interactions such as chelation and electrostatic interactions for ion exchange or ion pair formation [70]. It is found in the shells of crustaceans, the shells and skeletons of mollusks and krill, on the exoskeletons of arthropods, and in the cell walls of fungi. Depending on its source, three different crystalline polymorphic forms of chitin have been identified: α-chitin, the most abundant (shrimp and crab shells), β-chitin (squid pens) and γ-chitin (the stomach cuticles of cephalopoda) [1].

Zhang et al. [38] used 1-D negatively charged TEMPO-oxidized CNF and positively charged partly deacetylated chitin nanofiber to self-assemble a 3D biohybrid hydrogel (BHH) through electrostatic forces at room temperature. Then, they used freeze-drying to obtain a biohybrid aerogel (BHA). The specific surface area of the BHA was 54 m^2^/g. The amino and carboxyl groups on the surface provide adsorption sites. The maximum adsorption capacity for As^3+^ under the neutral pH conditions was 217 mg/g.

Chitosan is produced commercially by the deacetylation of chitin [71], and has strong ability to chelate heavy metal ions due to the amino and hydroxyl groups on its surface [72]. Rodrigues et al. [73] used chitosan-g-poly (acrylic acid) matrices filled with CNWs (cellulose nanowhiskers) to obtain hydrogel composites (Chitosan-g-poly(acrylic acid)/CNWs). The adsorption performance of hydrogels on Pb^2+^ and Cu^2+^ was investigated by controlling the amount of CMWS added. The highest adsorption of Pb^2+^ (818.4 mg/g) and Cu^2+^ (325.5 mg/g) was obtained within 30 min at pH 4.0 when using 20 mg of the hydrogel composite containing 10 *w*/*w*-% of CNWs. After washing with 0.1 mol/L HCl solution five times, the results showed that the adsorption capacity of Pb^2+^ and Cu^2+^ remained 89.3% and 81.8%, respectively.

Polyvinyl alcohol (PVA) is a low-cost polymer with desirable properties such as water solubility, biocompatibility, and biodegradability. The application of magnetic adsorbents technology has become a promising way to solve environmental problems [74]. Zhou et al. [75] used TEMPO to oxidize MCC to first obtain carboxylated cellulose nanofibrils (CCNFs) and then obtain CCNFs-filled magnetic chitosan hydrogel beads (m-CS/PVA/CCNFs) through an instantaneous gelation method. The carboxyl content of CCNFs was 0.94 mmol/g, and Pb^2+^ was supported by m-CS /PVA without CCNFs. Because the surface of m-CS/PVA/CCNFs contains carboxyl groups, the maximum adsorption capacity was 171 mg/g, which was higher than that of m-CS/PVA/117.6 mg/g. It removed Pb^2+^ mainly through amino chelation and carboxyl ion exchange. After four cycles with 0.01 M HNO_3_ regeneration, its adsorption efficiency remained 90%.

Alginate is a natural anionic polysaccharide, which is a linear chain of β-D-mannuronic acid (M units) and α-L-guluronic acid (G units) linked via 1,4-glycosidic bond, and is mainly obtained from brown algae and bacteria. Alginate can be mixed with nanocellulose to remove heavy ions in water thanks to its non-toxic and biodegradable properties, low cost, and rich carboxyl groups [76]. Hydrogels are considered as promising adsorbents for the removal of heavy metals from wastewater due to their many different functional groups and three-dimensional network structure [77,78,79]. Hu et al. [80] cross-linked carboxylated cellulose nanocrystal (CCN) and sodium alginate under the action of Ca^2+^ to obtain CCN-Alg hydrogel. It showed easy separation after adsorption, and had stronger mechanical strength and durability than pure sodium alginate beads. The proposed adsorption mechanisms could include electrostatic attraction and complexation. Within two hours, 76% of Pb^2+^ could be removed and adsorption equilibrium was quickly reached in three hours, with the highest adsorption capacity being 338.98 mg/g.

Carboxymethyl-chitosan (CMC) is a chitosan derivative containing abundant free hydroxyl (-OH), carboxyl (-COOH), and amine (-NH_2_) groups, and offers strong binding sites for heavy metal ions. Li et al. [81] prepared NSC gel beads using three carboxyl-containing materials (TEMPO-oxidized nanocellulose and sodium alginate and carboxymethylated chitosan under the action of Ca^2+^ crosslinking agent. The gel exhibited high efficiency for the adsorption of Cu^2+^ (169.94 mg/g) and Pb^2+^ (472.59 mg/g). The gel maintained a high adsorption capacity for Cu^2+^ (56 mg/g) and Pb^2+^ (245 mg/g) after five adsorption–desorption cycles.

Protein nanofibrils can be an eco-friendly strategy to engineer fully bio-based nanomaterials capable of removing hazardous Hg^2+^ from water sources. Silva et al. [82] dissolved lysozyme protein extracted from egg white in a solution containing 20 mmol/L glutamic acid and 5% (*v*/*v*) DES (choline chloride/acetic acid) to obtain lysozyme nanofibrils (LNFs), then mixed it with different proportions of CNFs to obtain dual nanofibrillar films (CNFs/LNFs). Combining the advantages of CNFs with high specific surface area and LNFs with a large number of peptide R-groups on their surface, these films have strong mechanical properties and binding capacity for Hg^2+^. The removal efficiency is pH-dependent, reaching a maximum of 99% (50 μg/L) after 24 h at a pH value close to the isoelectric point of the protein (pH = 11).

Activated carbon (AC) is widely used in the removal of heavy metal ions due to its high specific surface area and fast adsorption speed [83]. Septevani et al. [84] obtained cellulose-based EFB and lignin-rich black liquor from Oil Palm EFB by NaOH pulping method. AC was extracted from black liquor by Sari’s method [85]. NCS was obtained using the hydrolyzing cellulose with sulfuric acid, and NCP was obtained by phosphoric acid hydrolysis, then functionalized by AC to obtain NCS/AC or NCP/AC super-adsorbent. The SEM image revealed that AC was dispersed in the NCS and NCP matrix, forming a looser embedded network. Its adsorption mechanism was mainly the electrostatic attraction between adjacent hydroxyl groups and positively charged metal ions on the surface of the super-adsorbent, and NCS/AC’s maximum adsorption capacity for Pb^2+^ reached 24.94 mg/g.

### 3.2. Nanocellulose/Nitrogenous Polymer Composites

**Figure 8:**
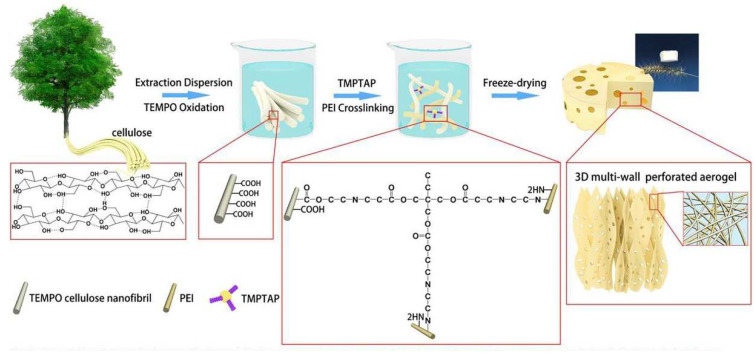
Schematic illustration and formation mechanism of crosslinked TO-CTP aerogel with 3D multi-wall perforated structure. Adapted with permission from [88]. Copyright 2019 Elsevier.

Amino groups have a strong chelating ability to heavy metal ions; thus, increasing the amino group content can increase the adsorption capacity. Because polyethyleneimine (PEI) has plenty of primary, secondary, and tertiary amines on the macromolecular chains, it is usually fabricated as a hydrogel or aerogel by crosslinking with an aldehyde or epoxy group to improve the adsorption capacity [86]. Tang et al. [87] obtained high amine group content (5.74 mmol/g) cellulose nanofibril/PEI aerogel beads (CGP1.3) with the help of a cross-linking agent of 3-glycidyloxypropyl) trimethoxy silane (GPTMS) by quickly freezing with liquid nitrogen and then freeze-drying. The maximum Cu^2+^ adsorption capacity reached 163.40 mg/g. Mo et al. [88] used a TO-CNF and Trimethylolpropane-tris-(2-methyl-1-aziridine) propionate (TMPTAP) ring-opening reaction at room temperature, then post-crosslinked PEI to obtain a 3D multi-wall structure TO-CNF/TMPTAP/PEI aerogel (TO-CTP) with pores (Figure 8). Because the aerogel had a large number of amino groups and oxygen-containing groups on the surface, its maximum adsorption capacity for Cu^2+^ was able to reach 485.44 mg/g. Moreover, after being treated with EDTA-2Na, the regenerated aerogels retained high removal efficiency for Cu^2+^ over four desorption-regeneration cycles.

In addition, glutaraldehyde (GA) is a common cross-linking agent [42], Zhang et al. [89] obtained TOCN by HCl hydrolysis and TEMPO oxidation, followed by cross-linking with PEI under the action of GA cross-linking agent, then freeze-drying and grinding to obtain TOCN-PEI adsorbent. Because of the linking between PEI and -COOH, the carboxyl content of TOCN-PEI decreased from 1.88 to 0.85 mmol/g, and its total amount of amino groups was 4.06 mmol/g. Its maximum capacity for Cu^2+^ reached 52.32 mg/g due to its abundant carboxyl and amino groups. After HCl cleaning, its adsorption capacity could remain able to reach 33 mg/g.

Li et al. [90] obtained a NFC solution through TEMPO oxidation, then achieved a physically-crosslinked network NFC/PEI composite hydrogel (NPH13, NPH22, NPH31) through electrostatic combination with PEI solutions of different weight ratios (1:3, 2:2, 3:1). After freeze-drying, NPA13, NPA22, NPA31 aerogels were obtained. The maximum specific surface area of the aerogels was 42.5 m^2^/g, and it had good shape recovery capacity. The electrostatic attraction and cation exchange between carboxyl and amino groups on Cu^2+^ and Pb^2+^ are the reasons for the high adsorption capacity of aerogels. The maximum adsorption capacities of NPA22 for Cu^2+^ and Pb^2+^ are 175.44 mg/g and 357.44 mg/g, respectively. After three adsorption/desorption cycles with EDTA solution, the adsorption capacity of NPAs was maintained at more than 90%.

Polyurethane (PU) has the advantages of high mechanical strength and stability, along with high specific surface area under different environmental conditions, and can be applied to the adsorption of heavy metal ions [91]. Hong et al. [92] filled different concentrations of CMCNFs (2, 3, 4 wt.%) in PU foam as filler to obtain PU/CMCNF (neat-PU, PU/CMCNF-2, 3, 4) foams. SEM images show that the surface of the composite foam material is rough and porous. The maximum adsorption capacities of CMCNF embedded in PU foam were found to be 78.7 mg/g and 216.1 mg/g for Cu^2+^ and Pb^2+^ removal, respectively, in PU/CMCNF-2, while PU/CMCNF-3 exhibited maximum removal capacity for Cd^2+^ (98 mg/g).

Polydopamine (PDA), formed by self-polymerization of dopamine (DA) in weak alkaline conditions, is rich in catechol and amine groups, which facilitate covalent conjugation or other noncovalent interactions with organic and inorganic materials [93]. Derami et al. [94] incorporated of PDA particles into a *Gluconacetobacter hansenii* broth under aerobic and static conditions. PDA particles were grown in situ on the BNC membrane. The catecholamine group on the surface of PDA particles has a strong affinity to lead ions. Adsorption of PDA/BNC was tested in a mixed solution of Pb^2+^, Cd^2+^. The PDA/BNC membrane removed 5.3 g of Pb^2+^ from water per square meter of the membrane area. The lowest yield was observed for Cd^2+^, with 2.1 g of the ions removed per square meter of the membrane area. After regeneration with 0.1 M sodium citrate solution, the regenerated membranes exhibited excellent contaminant removal efficiency even after ten cycles of filtration (with about 90% of the initial performance retained).

DA is known as a mussel adhesive protein-inspired molecule. Juntao et al. [95] introduced PDA particles into the surface of CNFs using a bio-inspired coating strategy for DA and then cross-linked PEI to form a porous aerogel (PDA-CNF-PEI). Its maximum adsorption capacity for Cu^2+^ reached 103.5 mg/g, and its porosity and density were 98.5% and 25 mg/cm^3^, respectively. When it was regenerated for four cycles using 0.1 M HCl treatment, its adsorption efficiency for Cu^2+^ remained more than 91%.

Polypyrrole (PPY) is an organic polymer formed by polymerization of the pyrrole ring (C_4_H_5_N). Extensive studies on this polymer justify its stability, low cost, and eco-friendly nature [96]. Shahnaz et al. [97] hydrolysed cellulose with sulfuric acid to obtain spherical NC and coupled it with PPY to obtain an adsorbent (NCPPY) for Cr^6+^ removal in water. Compared with 197 m^2^/g for NC, the specific surface area of NCPY was significantly increased to 488 m^2^/g. The maximum adsorption capacity of NCPPY for Cr^6+^ was 147.3 mg/g, possibly due to the -OH and -NH_2_ adsorption sites on its surface.

Electrospinning has been extensively applied to the preparation of nonwoven fabric-like films [98,99]. Due to preferable spinnability and wide range of inclusiveness of polyacrylonitrile (PAN), inorganic filler can be well distributed in the composites by wrapping and entanglement [100]. Because the nitrile groups on the surface of PAN can react with hydroxylamine in the aqueous solution at room temperature to form amidoxime groups, it has attracted widespread attention in the field of heavy metal ion removal.

Yang et al. [101] first obtained the CNFs through TEMPO oxidation, then modified the mercaptan group with cysteine, and then covered the PAN scaffold obtained by electrostatic spinning, and finally obtained the m-CNF membrane. The obtained oxidized CNFs through TEMPO oxidation were in turn grafted with cysteine to obtain the thiol group, which was embedded in the electrospun PAN scaffold. Ultra cellulose nanofibers have a large surface-to-volume ratio (~5 nm in diameter and a few hundred nanometers in length), which makes m-CNF membrane also have a large surface to volume ratio as well. The thiol Group is contained in m-CNF; the concentration is 0.9 mmol/g, and the maximum adsorption capacity for Cr^6+^ and Pb^2+^ is 87.5 mg/g and 137.7 mg/g, respectively. When the m-CNF membrane adsorbed by Cr^6+^ and Pb^2+^ was regenerated three times by the HCl (2 M) and EDTA (0.05 M) solutions, the m-CNF membrane continued to possess 93% of its original Cr^6+^ adsorption capacity and 95% of its original Pb^2+^ adsorption capacity.

### 3.3. Nanocellulose/Other Composites

Organic aerogels can exhibit many remarkable properties, including ultralow density, high porosity, high specific surface area, and excellent mechanical properties. Zheng et al. [102] prepared an aerogel from PVA solution and TOCNF under the action of a glutaraldehyde crosslinking agent. A comparison of the heavy metal ion adsorption performance of aerogels prepared from PVA solution with and without TOCNF showed that the maximum adsorption capacity of the PVA/CNF aerogel for Hg^2+^, Pb^2+^, Cu^2+^ and Ag^+^ was 157.5, 22, 110.6, and 24.5 mg/g, respectively, and was much higher than pure PVA aerogel. The carboxyl groups in the porous material showed electrostatic attraction and chelation for heavy metal ions, and it was possible to carry out adsorption of oils and organic solvents after silane superhydrophobic treatment.

Graphene oxide (GO) was used to remove heavy metal ions due to its high specific surface area and large number of functional oxygen groups that could provide active sites for heavy metal ions [103]. Yu et al. [104] used Fe^3+^ as a crosslinking agent, carboxymethyl cellulose nanofibril as a filler, and the wet-spinning method to obtain GO/CMCNF composite fiber (CF). The fiber exhibited enhanced tensile strength up to 452 MPa. Its maximum adsorption capacity for Pb^2+^ reached 99.0 mg/g by electrostatic attraction, ion exchange, and complexation of carboxyl groups and Pb^2+^.

Wood is one of the most abundant materials in nature, and has with excellent mechanical and anisotropy properties [20]. Mo et al. [105] prepared an aerogel with a biomimetic honeycomb architecture and specific covalent bonding networks following a wood-inspired method of directional freezing of liquid nitrogen. First, they mixed TCNF with different proportions of GO solution, then used TMPTAP for the ring-opening reaction to obtain a TCNF/TMPTAP/GO aerogel (TCTGAs). The maximum adsorption capacity of Pb^2+^ was 571 mg/g, the removal rate reached 100% within ten minutes for the selective adsorption of Pb^2+^.

Carbon dots (CDs) have been the subject of extensive research due to their chemical stability, excellent biocompatibility, non-toxicity, and colorful photoluminescence [106]. Guo et al. [107] obtained Carboxymethylated Cellulose Nanofibrils (CM-CNFs) from carboxymethylated Eucalyptus Kraft Pulp by sodium hydroxide/chloroacetic acid treatment and homogenization. The CM-CNFs were further modified with CDs based on a typical condensation reaction. The carboxyl group on the surface of CMCNFs reacts with the amino group on the surface of CDs to form -CO-NH and water. A series of fluorescent nanocellulosic hydrogels (FNH-1, 2, 3, 4, 5, 6) were prepared through radical polymerization of CM-CNF-CDs, AA (acrylic acid), AM (acrylamide) and MBA (N’, N-methylenebisacrylamide) using PPS (potassium persulfate) as the initiator. The resulting high content of amino, hydroxyl, and carboxyl groups provides adsorption sites, and the 3D network structure of the hydrogel promotes the adsorption of metal ions from the outside to the inside. The maximum adsorption capacity of the FNH-5 for Fe^3+^, Ba^2+^, Pb^2+^ and Cu^2+^ ions was tested, and the results were 769 mg/g, 212 mg/g, 2056 mg/g, and 1246 mg/g, respectively.

## 4. Nanocellulose/Inorganic Composite Adsorbents

The design of organic−inorganic hybrid materials currently plays a substantial role in the evaluation of innovative advanced materials.

### 4.1. Nanocellulose/Iron Composites

The magnetic separation technique is widely employed for separation and purification. Superbmagnetic ion materials include Ni, Co, Fe, Fe_2_O_3_, Fe_3_O_4_, Fe-Co, and Ni-Fe [108]. Among these, the Fe_3_O_4_ nano-ion is stable and widely used in culture mediums due to its low toxicity. BC (bacterial cellulose) is primarily synthesized from low molecular weight carbohydrates by *Gluconobacter* and *Acetobacter* [109]. Zhu et al. [108] synthesized BC from *Xylobacteria* by agitated fermentation method and biosynthesized a spherical Fe_3_O_4/_BC nanocomposite using a pH-controlled embedding method. The maximum adsorption capacities of Fe_3_O_4_/BC spheres for Pb^2+^, manganate (Mn^2+^), and Cr^3+^ were 65, 33, and 25 mg/g, respectively. Because the superparamagnetic spherical Fe_3_O_4_/BC nanocomposites are recycled using magnetic field separation, they can be utilized repeatedly. After recycling with 0.1 mol/L sodium citrate, the adsorption capacity of Fe_3_O_4_/BC spheres for the three ions decreased slightly.

**Figure 9:**
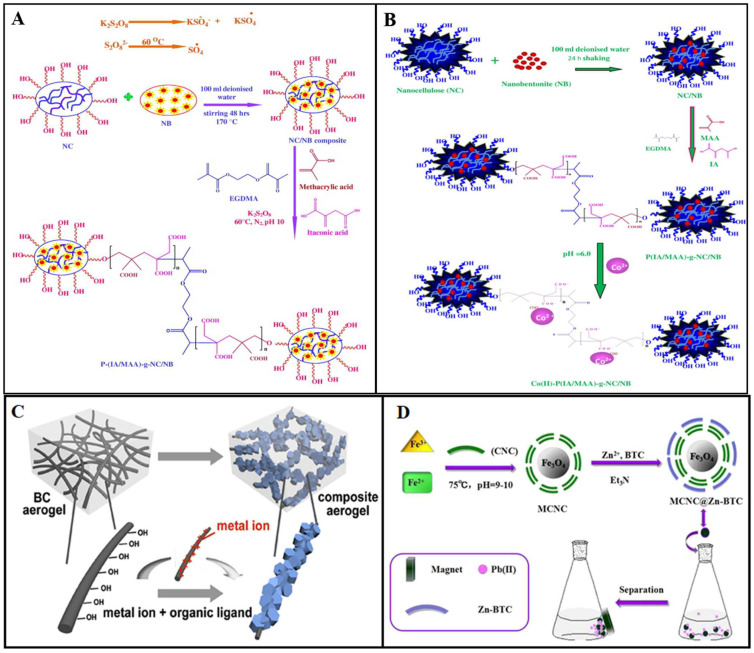
Synthesis of (**A**) P(IA/MAA)-g-NC/NB, (**B**) Co(Ⅱ) P(IA/MAA)-g-NC/NB, (**C**) BC@MOFs composite aerogels, and (**D**) MCNC@Zn-BTC. Adapted with permission from [116]. Copyright 2015 Elsevier. Adapted with permission from [117]. Copyright 2016 Elsevier. Adapted with permission from [118]. Copyright 2019 Elsevier. Adapted with permission from [119]. Copyright 2017 American Chemical Society.

Anirudhan et al. [110] used EGDMA (Ethyleneglycol dimethacrylate) as a crosslinking agent and K_2_S_2_O_8_ as a free radical initiator, which was grafted onto magnetite nanocellulose by itaconic acid and then further modified by 2-mercaptobenzamide to obtain a new type of thiol and carboxyl functionalized magnetite nanocellulose composite particle (P(MB-IA)-g-MNCC) for removal of Hg^2+^ from chlor-alkaline industrial wastewater (Figure 9A). MNCC was obtained by in situ growth of Fe_3_O_4_ on NC hydrolyzed by sulfuric acid. Hg^2+^ is removed by the ion exchange of carboxyl groups and the complexation of thiol groups, and its maximum adsorption capacity is 240.0 mg/g. They used the same adsorbent to remove Cd^2+^ [111] (Figure 9B)and Co^2+^ [112], with a maximum adsorption capacity of 262.27 mg/g and 349.62 mg/g, respectively.

Prussian blue (PB), with the formula Fe_4_^III^[Fe^II^(CN)_6_]_3_, contains polar Fe^II^−C−N−Fe^III^ units [113] and is considered to be a promising material thanks to its excellent adsorption properties and high selectivity for Cs ions [114]. However, the use of PB nanoparticles to remove Cs from a radioactive waste solution is limited by separation problems. Eun et al. [115] first used Fe^3+^ as a crosslinking agent to crosslink CMCNF membrane, then used Fe^3+^ ions as a precursor to prepare in-situ growth of PB. The PB nanoparticles, which exhibit an irregular morphology, densely cover the surface of the PB-CMCNF sample. The removal mechanism of Cs^3+^ due to ion exchange between Cs and K. The 0.5 M-PB-CMCNF membranes exhibited excellent Cs^3+^ uptakes of approximately 130 mg/g_PB-CMCNF_.

### 4.2. Nanocellulose /MoS_2_ Composites

MoS_2_ is a transition metal hydrogen disulfide, a layered structure composed of stacked two-dimensional nanosheets [120]; this film displays a large number of sulfide (S^2−^) sites, making it easy to bind heavy metal ions by electrostatic, hydrophobic, or chemical complexation interactions.

Ferreira-Neto et al. [121] prepared a hybrid functional photocatalyst by supporting MoS_2_ nanostructures on flexible self-standing macro-mesoporous bacterial nanocellulose aerogel membrane. Its specific surface area and pore volume were 97–137 m^2^/g and 0.28–0.36 cm^3^/g, respectively. Cr^6+^ was removed through an adsorptive–photocatalytic mechanism, with MoS_2_ showing effective visible light photoactivity and removing Cr^6+^ ions (88% removal within 120 min, K*obs* (apparent rate constant) = 0.0012 min^−1^) in photo-assisted inflow.

### 4.3. Clay/Nanocellulose Composites

Clay’s high specific surface area, high cation exchange capacity, and incredible physical and chemical stability can enhance heavy metal ion adsorption [122]. Hydroxyapatite (CHA) is an effective adsorbent material due to its capability for simultaneous removal of cationic and anionic contaminants from water [123]. Sanna et al. [124] used NCC as a template, with CHA particles and bentonite clay dispersed in a cellulose matrix to obtain CHA-BENT-NCC particles. The maximum adsorption capacity for Ni^2+^ and Cd^2+^ was 22.96 mmol/g, 9.71 mmol/g, respectively. CHA-BENT-NCC can be regenerated by 0.1 M HNO_3_. After five cycles, the adsorption capacity of CHA-BENT-NCC was decreased from 97% to 74% for Cd^2+^ and from 98% to 80% for Ni^2+^.

Anirudhan et al. [116] used ethylene glycol dimethacrylate (EGDMA) as a crosslinking agent, potassium peroxydisulfate (KPS) as the initiator, and modified methacrylic acid (MAA) and itaconic acid (IA) in nanocellulose/nanobentonite (NC/NB) composite to obtain PIA/MAA-g-NC/NB. U^6+^ was removed by ion exchange of carboxyl functional groups. With the increase of initial U^6+^ concentration from 100 to 250 mg/L, the adsorption capacity was increased from 49.73 to 121.02 mg/g. Simulated nuclear industry wastewater was used for practical efficiency and effectiveness tests, and 0.45 g/L adsorbent was observed to be sufficient for the complete removal of U^6+^. After six adsorption–desorption cycles with 0.1 M HCl, a slight decrease in adsorption capacity was observed, from 94.22% to 89.60%. When using the same adsorbent for the removal of Co^2+^ [117], the maximum adsorption capacity was 350.8 mg/g. After washing with 0.1 M HCl solution and six adsorption cycles, there was little loss (from 99.15% to 88.9%), meaning that the adsorbent could be applied in nuclear industrial wastewater.

## 5. Organic/Inorganic/Nanocellulose Composites

Hosseini et al. [125] prepared CNFs from date palm tree waste, cellulose nanofibril cryogel modified with 10% GO, and 10% Fe_3_O_4_ nanoparticle as filler (CNFs/GO/Fe_3_O_4_), prepared by facile freeze-drying methodology. CNF can enhance the mechanical strength and adsorption capacity of adsorbents. The CNFs/GO/Fe_3_O_4_ cryogel had a low density of 0.0139 g/cm^3^, an ultra-porosity of 99.46%, and appropriate specific surface area (S_BET_ = 55 m^2^/g). The maximum adsorption capacity of the CNFs/GO/Fe_3_O_4_ cryogels for Pb^2+^, Hg^2+^, Cr^6+^ at 298 ± 1 K were 126.58, 36.7, and 73.52 mg/g, respectively. After four cycles of 1 M HCl, the removal rates of Pb^2+^, Cr^6+^ and Hg^2+^ were able to reach 97.3, 96.51, and 88.5% of the initial run.

Shahnaz et al. [126] hydrolyzed cellulose with sulfuric acid and oxidized sodium periodate to obtain dialdehyde-based nanocellulose (DANC). Bentonite was converted into NB (nanobentonite) under ultrasound. Chitosan was modified with chloroacetic acid to obtain carboxymethyl chitosan (CMC). CMC, DANC, and NB were mixed to obtain hydrogel slurry, which was freeze-dried to obtain the NB incorporated dialdehyde nanocellulose-carboxymethyl chitosan aerogel (NBNC). The response surface methodology (RSM) method was used to analyze the optimal reaction conditions of NBNC to heavy metal ions. The adsorption capacity for Cr^6+^, Co^3+^, and Cu^2+^ were 2749.68, 916.65, and 1937.49 mg/g, respectively.

MOFs (metal-organic framework) are a new kind of porous crystalline materials [127], which are formed by the interaction between inorganic metal ions (metal clusters) and organic ligands [128]. Due to their high specific surface area, adjustable pore volume, and pore size distribution, it can be used for heavy metal ion adsorption. ZIF-8 (Zeolitic Imidazolate Framework-8) is a typical MOF material composed of Zn metal atoms and 2-methylimidazole [129]. Ma et al. [118] grew ZIF-8 on BC aerogel by an in-situ growth method and obtained a BC@ZIF-8 composite aerogel (Figure 9C). SEM images showed that the aerogel was composed of BC and ZIF-8 nanoparticles, and XRD results showed that the ZIF-8 and BC@ZIF-8 had similar crystalline structures. The maximum adsorption capacities of BC@ZIF-8 aerogel for Pb^2+^ and Cd^2+^ were 390 and 220 mg/g, respectively.

Wang et al. [119] synthesized magnetic CNC (MCNC) with CNC and Fe_3_O_4_, then synthesized MCNC@Zn-BTC adsorbent by reaction of Zn and homo-phenic acid with MCNC through a simple mechanical stirring method to adsorb Pb^2+^ in water. Scanning electron microscopy showed that Zn-BTC presented a perfect columnar crystal structure, and the surface of MCNC@Zn-BTC showed a beaded crystal structure (Figure 9D), indicating that Zn-BTC successfully covered the surface of MCNC. The adsorption results showed that the maximum adsorption capacity of Pb^2+^ reached 558.66 mg/g at 298.2 K, and adsorption equilibrium was reached within 30 min. After five adsorption–desorption cycles, the adsorption capacity remained able to reach more than 80%.

## 6. Conclusions

As an emerging materials platform for the removal of heavy metal ions, nanocellulose-based adsorbents offer many advantages, however, there are challenges that need to be addressed appropriately in the future. For instance, given the strong negative ion groups, nanocellulose-based adsorbents have a high electrostatic attractiveness, offering desired adsorption sites for heavy metal ions. However, negative hydrophilic ion groups diminish the hydrophobic capacity and stability of water adsorbents. Potential solutions include, (though are not limited to) modification of nanocellulose or integration of assembly processes to improve cross-linking behaviours between nanocellulose and other furniture.

Surface modifications of nanocellulose, such as, oxidation, phosphorylation, and amination, could promote the adsorbing sites of nanocellulose-based adsorbents, although this would likely result in a rapid decrease in their desorption ability. In order to increase the recycling times for adsorbents, more acidic washing is necessary, which causes environment damage. To obtain high desorption capacities, it is firstly necessary to find materials that have different binding affinities for heavy metal ions, then assemble these substrates into multilayer 2D/3D nanocellulose-based adsorbent aerogels or composites, and finally prepare the nanocellulose based adsorbents with high adsorption/desorption ability.

## 7. Future Direction and Beyond Limitations

**Figure 10:**
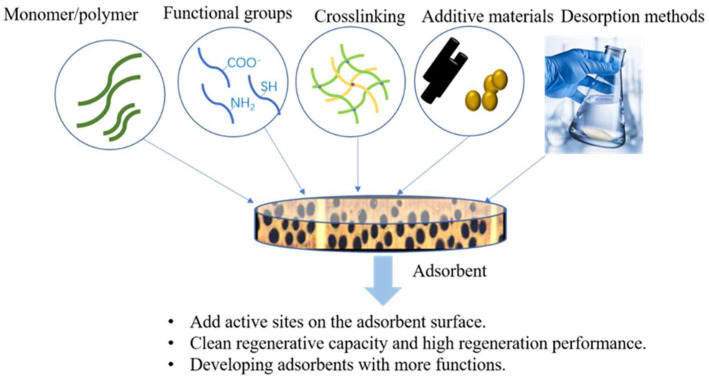
By regulating key elements, including monomers/polymers with different functional groups, and cross-linking types and desorption methods promote nanocellulose-based adsorbents with many advanced properties that excel in heavy ion removal applications.

To meet the requirements of recycling, selective adsorption, or desorption of different heavy metal ions, precisely controlled assemblies of nanocellulose-based adsorbents with tailorable hydrophilicities and mechanical properties are desired for industrial applications. For example, in order to optimize the assembly process, crosslinking agent, and function, additive types of adsorbent networks have to be properly selected to modulate the suitable porous structures and adsorption capacities (Figure 10). In addition, computational modeling and advanced in situ characterizations are beneficial for providing further guidance to researchers in the rational design of cellulose-based adsorbents for heavy metal ion removal.

## Figures and Tables

**Table 1:** Maximum concentrations and hazards of heavy metal ions in drinking water, data from US EPA.

Pollutants	MCLG (Maximum Pollutant Concentration Index) (mg/L)	Potential Health Effects
Sb	0.006	Increases blood cholesterol and decreases the amount of glucose in the blood
As	/	Damage the skin and increase the risk of cancer
Ba	2	Elevation of blood pressure
Cd	0.005	Kidney injury
Cr	0.1	/
Cu	1.3	Gastrointestinal pain, liver or kidney injury
Pb	0	Delayed physical or mental development
Inorganic mercury	0.002	Kidney injury
